# Mutant p53 and ETS2, a Tale of Reciprocity

**DOI:** 10.3389/fonc.2016.00035

**Published:** 2016-02-18

**Authors:** Luis Alfonso Martinez

**Affiliations:** ^1^Department of Pathology, Stony Brook Cancer Center, Stony Brook University, Stony Brook, NY, USA

**Keywords:** p53, Ets2, cancer, tumor suppressor protein p53, transcription factors

## Abstract

TP53 is one of the most frequently inactivated tumor suppressor genes in human cancer. However, unlike other tumor suppressor genes whose expression is lost, TP53 is usually inactivated as a result of a single nucleotide change within the coding region. Typically, these single nucleotide mutations result in a codon change that creates an amino acid substitution. Thus, unlike other tumor suppressor genes whose expression is lost due to genetic or epigenetic changes, the p53 gene primarily suffers missense mutations, and therefore, the cells retain and express a mutant form of the p53 protein (mtp53). It is now well established that mtp53 contributes to tumor development through its gain-of-function (GOF) activities. These GOF activities can arise from novel protein–protein interactions that can either disable other tumor suppressors (e.g., p63 and p73) or enable oncogenes such as ETS2, an ETS family member. In this review, I will focus on the identification of the mtp53/ETS2 complex and outline the diverse activities that this transcriptional regulatory complex controls to promote cancer.

## Simultaneous Inactivation of Wild-Type p53’s Tumor Suppressor Activity and Activation of Mutant p53’s GOF Activities

The majority of mutations in the p53 gene cluster within the region that encodes the DNA-binding domain. Some of these mutations alter the overall conformation of the protein (referred to as structural mutants), while other mutations do not alter the structure but instead change an amino acid that is critical for DNA binding (referred to as DNA contact mutants) (1, 2). These mutations typically give rise to mtp53 proteins that have lost the capacity to bind to the wild-type p53 (WTp53) consensus binding site and are thus unable to associate with WTp53 response elements in the genome and therefore unable to regulate WTp53 target genes. However, mtp53 is present on the promoters of various genes and is able to regulate their expression (1, 2). These observations indicate that despite having lost its WTp53 sequence-specific DNA-binding activity, mtp53 is still capable of acting like a transcription factor.

Initial studies on mtp53 relied on the overexpression of its cDNA in cells that were p53 null (3). In these studies, it was shown that mtp53 functions in a manner that is diametrically opposed to the tumor suppressor functions of WTp53. Instead of suppressing cancer or simply acting like an inert protein (due to its mutational inactivation), the mtp53 protein exhibited GOF activities, which allowed it to promote growth and tumorigenesis (3). From these studies, it became apparent that mtp53 can function as an oncogene, and these GOF activities were most apparent when the mtp53 harboring cells were challenged, for example, with proapoptotic stimuli (1, 2). The advent of siRNA technology permitted endogenous mtp53 to be suppressed in cells, which resulted in apoptosis (4). These data argued that mtp53 is actively engaged in promoting cell survival, and thus, cells harboring these mutant proteins exhibit an addiction to them. Addiction to mtp53 has since been demonstrated by various groups and has recently been genetically confirmed in mice (5, 6). It is important to note from these latter studies *in vivo* that early intervention delays the tumorigenesis process, which could indicate that mtp53 is required to drive the carcinogenic pathway early on and possibly that addiction to this oncogene is an early occurring event, even in cells that are not yet “transformed.” This latter view is supported by the fact that siRNA knockdown of mtp53 in non-transformed/non-tumorigenic Li–Fraumeni fibroblasts resulted in apoptosis, indicating that these cells exhibited oncogene addiction (4).

## Mechanistic Basis for Mutant p53’s Transcriptional Regulatory Oncogenic Functions

To investigate the basis of its GOF activities, we and others have performed genome-wide analysis of mtp53 binding, and through bioinformatics and biochemical analysis have determined that mtp53 can be recruited to promoters *via* interactions with other transcription factors (7–11). Many of these transcription factors that bind to mtp53 have also been shown to interact with WTp53 (E2F1, NF-Y, VDR, ETS1, ETS2, and SP1), although there are some discrepancies among different studies (7–12). For example, one of the earliest studies to show that mtp53 regulates gene expression *via* the recruitment mechanism was on the regulation of the MDR1 promoter (13). In this study, it was reported that ETS1 can only interact with mtp53 and not with WTp53 (13). Other studies had shown that WTp53 can also interact with ETS1 (14, 15). ETS1 has been shown to be required for the transcriptional regulatory activity of WTp53 (16). Thus, it appears that both the oncogenic and tumor suppressor forms of p53 might rely on the ETS factors. We and others have reported that WTp53 at best poorly interacts with ETS1 (10, 11). What is the basis for the discrepancies between studies? It is possible that there might be tissue-specific or stress-dependent conditions that permit WTp53 to interact with ETS family members, although some studies were conducted *in vitro*. Additionally, WTp53 has been shown to undergo conformational changes during cell cycle progression. In this case, the protein can adapt mtp53-like conformational attributes (17–20). Does this reflect a conformational speciation of p53, where depending on cellular growth conditions or stress, p53 can adapt to different conformations that transiently increase its binding partners’ repertoire. If this is the case, perhaps WTp53 can oscillate between different conformational species, a “minor” form of which is able to interact with ETS1. In contrast, mtp53 is locked in the minor form conformation that allows it to bind extensively to other protein partners including ETS1.

## The E26 Transformation-Specific Motif is Overrepresented in Mutant p53 Occupied Promoters

Chip-Chip and Chip-Seq data revealed that approximately 50% of the promoters occupied by mtp53 contain ETS-binding sites, suggesting that the association with ETS proteins is a prominent mechanism by which mtp53 regulates gene expression (10, 11). Mtp53 has been shown to associate with promoter regions of genes in an ETS2-dependent manner (10, 11). Importantly, these mtp53-bound genomic regions do not have a WTp53 response element, indicating that the mtp53 protein is not associating with these targets through residual activity of its DNA-binding domain (10, 11). Additionally, these mtp53 target genes do not overlap with WTp53 target genes that are identified through a similar genome-wide analysis, further demonstrating that mtp53 associates with these promoters in a completely novel manner (10, 11). Although the interaction with ETS1 might be important for the regulation of some mtp53 target genes, side-by-side comparison using recombinant proteins revealed that mtp53 preferentially associates with ETS2, another ETS family member (10). Both the structural and DNA contact p53 mutants interacted with ETS1, albeit with seemingly less affinity (10). It was also noted that the structural mutant (R175H) bound ETS1 better than the DNA contact (R248W) mutant. Importantly, all structural and DNA contact p53 mutants that have been tested thus far interact with ETS2 (10, 11). Moreover, whereas ETS1 knockdown generally has no effect on mtp53 target gene expression, ETS2 knockdown recapitulates the changes in gene expression that occur upon mtp53 knockdown (10, 11, 21, 22). Nevertheless, the observation that ETS2 interacts with various mtp53 (R175H, R248Q, R248W, R249S, R273H, R273L, and R280K) suggests that by coupling with ETS2, different mtp53 proteins are able to exert oncogenic activities through a common platform. The mtp53 proteins that have been tested correspond to the “hot-spot” mutations. It will be of interest to determine if proteins generated by missense mutations that are outside the region encompassed by the cluster of hot-spot mutations also interact with ETS2. However, further analysis is required to determine if all cancer-associated p53 mutants interact with ETS2. This is an important analysis because it has long been established that there are differences in the oncogenic potency of distinct p53 mutants, which might be related to their affinity for ETS2 or even other ETS factors. In this regard, it will be important to determine if p53 mutants that are more active than WTp53 in transcriptional and cell killing assays also interact with ETS2 (23).

It is of particular interest to note that ETS2 binds to the tetramerization domain of p53, which is thought to be functionally intact in both WTp53 and mtp53 (10). The question of how ETS2 distinguishes between mtp53 and WTp53 is further highlighted by the fact that some p53 mutants are considered to have subtle changes in their structure but are otherwise conformationally similar to WTp53. Intriguingly, it has been suggested that because WTp53 is actively engaged in sampling DNA sequences throughout the genome, it might not be able to interact with ETS2 (24). A corollary of this model would be that when WTp53 is associated with DNA, it might alter its structure in a manner that is incompatible with binding to ETS2. However, both overexpression and *in vitro* studies using recombinant proteins failed to show a strong interaction between WTp53 and ETS2. In the overexpression experiments, it seems unlikely that all of the transfected WTp53 protein is bound to DNA and thus cannot bind ETS2. Furthermore, the observation that the WTp53 and ETS2 purified proteins do not interact *in vitro* casts doubt on, yet does not eliminate, the possibility that the structural changes due to DNA binding by WTp53 prevent its interaction with ETS2 (10).

## Domain Requirements for Mutant p53’s Transcriptionally Dependent GOF

Since WTp53 has potent transactivation domains in its N-terminus, this raises the possibility that mtp53 can also utilize them to regulate gene expression. In support of this, mutation of the N-terminal transactivation domain of mtp53 eliminated its ability to activate the MDR-1 promoter and enhances tumorigenic potential (25). A similar conclusion was drawn in another study, in which the N-terminus was shown to be required for the transactivation activity of mtp53 (26). In contrast, it was observed that the C-terminus was required for mtp53 to promote tumorigenicity (26). Likewise, an intact transactivation domain appears to be required for mtp53 to promote chemotherapy resistance (27, 28). It appears that mtp53 may be able to mediate GOF activities using different domains. The precise mechanism(s) by which these mutations disable its oncogenic activity is not well understood; however, it has been reported that mutation of the transactivation domain in mtp53 disrupts its interaction with ETS1 (13).

An mtp53, in which the transactivation domain was mutated, was still capable of activating the promoter of one of its target genes, TDP2, in a luciferase assay (10). An mtp53 mutant lacking the C-terminus, which eliminates the interaction with ETS2, was unable to activate this promoter (10). However, p53 contains two transactivation domains, and mutation of both domains prevents mtp53 from disrupting mammary tissue architecture *in vitro* (29). These observations suggest that both transactivation domains may be required for mtp53 to exert its GOF. However, in the latter case, mtp53 was mediating its effects through an interaction with SREBP transcription factor, which raises the possibility that the domains required for GOF are dependent on the particular binding partner for mtp53.

## Mutant p53 Takes Care of Its Partner

**Figure 1:**
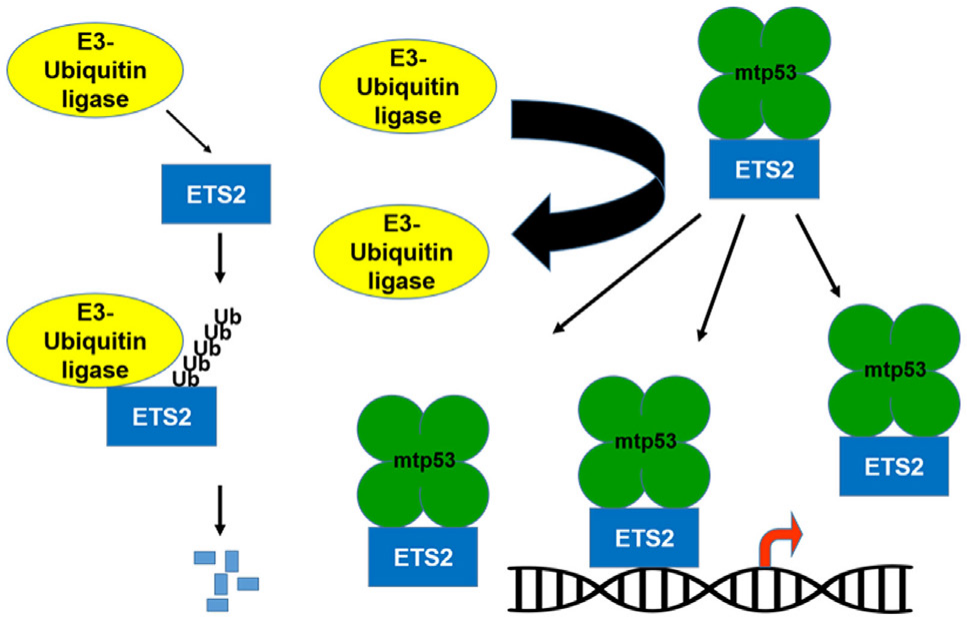
**Mutant p53 protects ETS2 from degradation**. ETS2 is a labile protein with a short half-life. An E3-ubiquitin ligase binds to ETS2 and promotes its ubiquitin-dependent degradation. In the presence of mtp53, ETS2 is not ubiquitinated and becomes stable, which increases its abundance allowing it to recruit mtp53 to ETS target genes.

**Figure 2:**
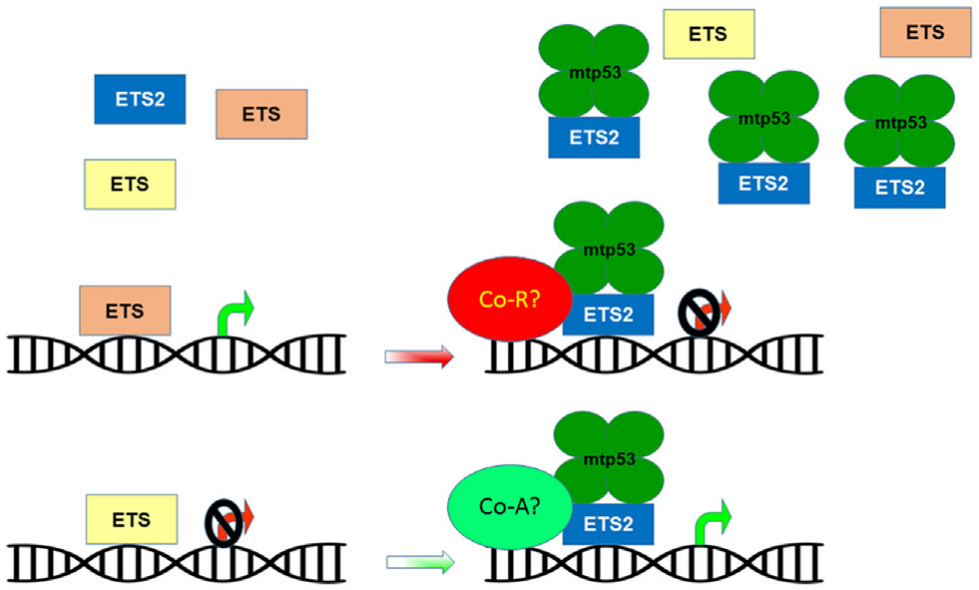
**Mutant p53 disrupts ETS family target gene regulation**. The ETS family of transcription factors share multiple target genes based on their ability to bind a common DNA motif. These shared targets can be regulated in a cooperative or opposing manner in order to maintain homeostatic control of gene expression. The presence of mtp53 causes ETS2 to accumulate and outcompete other ETS family members for binding to target genes, potentially altering their repression/activation by recruiting co-repressors (Co-R) or co-activators (Co-A).

If the transcriptional activation domain of mtp53 is not required for activation of gene expression, what is mtp53 contributing to this transcriptional regulatory complex? Importantly, mtp53 can protect ETS2 from ubiquitin-dependent degradation, which raises the possibility that by increasing ETS2 abundance, mtp53 disrupts the balance between activator/repressor ETS family members, favoring the presence of mtp53/ETS2 on promoter targets (Figures 1 and 2) (10, 30). Among the different mtp53 interacting proteins, ETS2 appears to be unique in that mtp53 protects it from degradation. There are various different proteins currently implicated in promoting ETS2 degradation including Cdh1/Fzr1, the adaptor protein for the APC/cyclosome complex; Cul4a, a subunit of the SCF ubiquitin ligase complex; the E3-ubiquitin ligase, COP1/RFWD2; and CDK10 (31–34). Further work is required to establish how mtp53 interferes with the function of one or all of these proteins to stabilize ETS2.

## Mutant p53’s Partnering with ETS2 Confers It Access to a Multitude of Oncogenic and Tumor Suppressive Transcriptional Targets

The ETS family of winged helix-turn-helix transcription factors consists of 28 family members that share a highly conserved DNA-binding domain, referred to as the ETS domain (30). The ETS domain permits all the family members to bind in a sequence-specific manner to a common core motif, GGAA [called the ETS-binding site (EBS)], and thus, they share many transcriptional targets (30). This overlapping set of targets raises the question of why there is such redundancy in gene regulation. The perplexing redundancy in gene targets is explained by the fact that ETS family members largely exhibit tissue-specific expression and that they can play both cooperative and/or opposing roles in regulating gene expression (30, 35). As such, only a subset of ETS family members are expressed in a given tissue, and the particular ETS family member that is occupying a particular binding site is dependent on extracellular cues (36). Ectopic expression of oncogenic ETS proteins can functionally substitute for activation of the Ras/MAPK pathway, implying that control of oncogenic ETS factor levels is imperative to prevent neoplastic transformation (37). The ETS family regulates diverse cellular activities including apoptosis, angiogenesis, cell growth, adhesion, migration/invasion, the extracellular matrix, and other transcription factors (30). Thus, by interacting with ETS2, mtp53 can hijack the ETS transcriptional repertoire and control many of these processes to promote cancer.

As can be surmised from the various cellular activities that the ETS family controls, the ETS family members can function as either oncogenes or tumor suppressor genes, and sometimes the context determines their role in promoting or suppressing cancer. For example, ELF3 is frequently mutated in cervical, mucinous ovarian, and biliary tract cancers (38–40). Ectopic expression of wild-type ELF3 suppresses cell growth of biliary tract and cervical cancer cells suggesting a tumor suppressor role in these cancers (38, 39). In contrast, ELF3 has been shown to function as an oncogene in colorectal and prostate cancer (41, 42). SPDEF, a prostate epithelium-specific ETS transcription factor, suppresses prostate cancer progression and metastasis (43–45). Knockdown of another ETS family member, ESE3/EHF, in normal prostate cells resulted in the acquisition of mesenchymal and stem-like characteristics (46). Chromosomal rearrangements have been shown to give rise to oncogenic gene fusions for multiple ETS family members including ERG, ETV1, ETV4, ETV5, ETV6, ELK4, and FLI1 (47). Importantly, ETS2 itself has been shown to be deleted in a subset of prostate cancers and to have a growth inhibitory function, suggesting that it is a tumor suppressor gene in this tissue. In addition, a transgenic mouse overexpressing ETS2 in the thymus had increased p53-dependent apoptosis (48). Previously, it has been shown that Ets2 dosage can impact tumor development in the APC^Min^ mouse model (49). Mice carrying extra copies of ETS2 were protected from tumor development, whereas ETS2 heterozygous mice exhibited higher cancer frequency (49). It is interesting to note that in the context of mutant p53 harboring cells, ETS2 abundance is increased yet it appears to function as an oncogene. Given that ETS2 has been shown to activate p53-dependent apoptosis, it is possible that the loss of wild-type p53 provides a permissive environment for ETS2 to have oncogenic functions. Taken together, the ability of mutant p53 to stabilize ETS2 and to utilize it to regulate gene expression constitutes a novel mechanism by which an ETS family member promotes cancer.

## Altered Target Selection vs. Amplified Regulation

There are various aspects of the mtp53/ETS2 regulatory complex that remain to be explored. For example, are the genes regulated by mtp53/ETS2 different from the ones regulated by ETS2 alone? Additionally, does the mtp53/ETS2 interaction alter the regulation (i.e., activation or repression) of these target genes? Since ETS2 is induced by growth factor receptor pathways, does mtp53 unlink it from mitogenic signaling and thereby produce a constitutively active ETS2. A clue comes from the observation that many of the mtp53/ETS2 target genes are controlled by ETS2 in cells lacking p53 (21, 22). This suggests that mtp53 is not altering the spectrum of genes that ETS2 controls but rather further enhancing their expression. For example, mtp53 was shown to upregulate nucleotide metabolism genes (NMG) expression by associating with their promoters, and suppression of mtp53 or ETS2 reduced their expression (22). In cells lacking mtp53 (i.e., either containing WTp53 or lacking p53 altogether), ETS2 knockdown reduced the expression of these target genes (22). Introduction of mtp53 increased ETS2 protein and NMG expression to levels higher than in cells lacking mtp53 (22). These data reinforce the notion that the mtp53/ETS2 complex upregulates NMG expression. Again, in this situation, ETS2 knockdown in cells ectopically expressing mtp53 reduced NMG expression, despite the fact that it did not affect mtp53 levels (22). These data suggest that the mtp53-mediated aberrant accumulation of ETS2 can enhance the expression of ETS2 target genes. In addition, removal of mitogens (*via* serum deprivation) results in reduced expression of the NMG in cells lacking mtp53, yet has no effect in cells expressing mtp53 (22). This observation raises the possibility that mtp53 is capable of superseding the mitogenic control of ETS2 function. Whether mtp53 is obviating intrinsic ETS2 auto-inhibitory activity or simply increasing its abundance, or both, to enhance ETS2 function requires further investigation (50).

## Future Directions

The cooperation between mtp53 and ETS2 to regulate gene expression is well established *in vitro*, but the extent to which these two work together to promote tumorigenesis *in vivo* is still not known. Furthermore, there is circumstantial evidence that mtp53’s GOF depends on several domains, and thus, it will be important to dissect these different domains *in vivo* to determine if one of these is dominant or whether the GOF is mediated by the action of multiple domains in mtp53.

## Author Contributions

The author confirms being the sole contributor of this work and approved it for publication.

## Conflict of Interest Statement

The author declares that the research was conducted in the absence of any commercial or financial relationships that could be construed as a potential conflict of interest.
